# Rapid evolution in response to introduced predators I: rates and patterns of morphological and life-history trait divergence

**DOI:** 10.1186/1471-2148-7-22

**Published:** 2007-02-14

**Authors:** Debra L Fisk, Leigh C Latta, Roland A Knapp, Michael E Pfrender

**Affiliations:** 1 Department of Biology, 5305 Old Main Hill Road, Utah State University, Logan, UT 84322, USA; 2 Sierra Nevada Aquatic Research Laboratory, University of California, HCR 79, Box 198, Mammoth Lakes, CA 93546, USA

## Abstract

**Background:**

Introduced species can have profound effects on native species, communities, and ecosystems, and have caused extinctions or declines in native species globally. We examined the evolutionary response of native zooplankton populations to the introduction of non-native salmonids in alpine lakes in the Sierra Nevada of California, USA. We compared morphological and life-history traits in populations of *Daphnia *with a known history of introduced salmonids and populations that have no history of salmonid introductions.

**Results:**

Our results show that *Daphnia *populations co-existing with fish have undergone rapid adaptive reductions in body size and in the timing of reproduction. Size-related traits decreased by up to 13 percent in response to introduced fish. Rates of evolutionary change are as high as 4,238 darwins (0.036 haldanes).

**Conclusion:**

Species introductions into aquatic habitats can dramatically alter the selective environment of native species leading to a rapid evolutionary response. Knowledge of the rates and limits of adaptation is an important component of understanding the long-term effects of alterations in the species composition of communities. We discuss the evolutionary consequences of species introductions and compare the rate of evolution observed in the Sierra Nevada *Daphnia *to published estimates of evolutionary change in ecological timescales.

## Background

Contemporary natural populations are faced with an unprecedented array of challenges as a direct result of anthropogenic influences on the environment. These anthropogenic influences may lead to environmental alterations that exceed the rate of change contemporary natural populations have historically experienced. Rapidly changing environmental conditions place natural populations at risk of extinction, and the current rate of extinction is increasing [1,2]. When rapid environmental change occurs, the likelihood of population persistence can be enhanced through a number of mechanisms including dispersal [3], phenotypic plasticity [4], and genetic adaptation [5]. Thus, understanding the dynamics of adaptive responses to changing environments is of central importance to evolutionary biologists, ecologists, and conservation biologists.

One of the most ubiquitous anthropogenic influences on ecosystems is the introduction of non-native species. The evolutionary component of species invasions has been the growing focus of studies on the adaptation of introduced species in a novel environment and the evolutionary consequences for native species in invaded communities [6,7]. Intentional and accidental introductions of non-native species into naive communities in many cases comprise a particularly dramatic environmental change that can result in rapid adaptive change [5,8]. While most examples of rapid adaptation stem from the ecological and evolutionary response of invaders in novel environments [7], native species within an invaded habitat frequently exhibit adaptive responses to introductions of novel species [9-12]. In special cases where the introduced species is a novel predator, native prey species can experience abrupt changes in the intensity or direction of natural selection [5,13]. In order to persist during changes in the selective environment, native prey populations require rapid evolution of behaviour, morphology, or life-history traits [14]. Examples of specific consequences of altered selection regimes arising from introduced predators include the acquisition of alarm responses [15], changes in patterns of diel vertical migration patterns [16], altered habitat use [17], increased escape ability [18], and reductions in body size and age at maturity [13].

Planktivorous fish are commonly introduced into freshwater systems for recreational fishing purposes [19-21] and can pose particularly strong selective challenges for native planktonic invertebrate populations. Many fish species, including salmonids, are highly efficient visual predators that selectively feed upon larger, more conspicuous zooplankton [22-25]. A primary prey item for fish, *Daphnia*, are especially vulnerable to fish predation due to their relatively large body sizes and poor swimming abilities [26]. However, *Daphnia *are capable of rapid evolutionary response to changing environmental conditions [27,28], and they have long been used as a model ecological system to study the consequences of changing selective challenges arising from fish introductions [29-32]. Novel size-selective predation on native *Daphnia *populations has precipitated rapid evolution in traits primarily related to detection avoidance, including alterations in patterns of diel vertical migration (DVM) [16,33] and reduced body size [23,30,32]. Fish predation can also result in changes in life-history traits, such as clutch size and growth rate [34].

The alpine lakes throughout the Sierra Nevada in eastern California, USA are ideally suited for investigation of the process of rapid adaptive evolution in response to abrupt changes in the selective environment. The lakes of the Sierra Nevada have been the subjects of extensive ecological study [35-37] in large part because the history of fish introductions is well documented. Nearly all lakes in the Sierra Nevada were historically fishless but the majority were stocked with one or more species of trout during the past century [36,37]. Fish presence/absence is often the best predictor of zooplankton species composition (*e.g.*, when fish are present in a lake, larger zooplankton are usually absent [38]), and the introduction of non-native fish into many historically fishless lakes has led to the extirpation of vulnerable invertebrate species [35-37].

The *Daphnia *community in alpine lakes of the Sierra Nevada is characterized by a single large-bodied, highly pigmented species, *Daphnia melanica *(genetic analyses indicate that the *Daphnia middendorffiana *referenced in earlier papers [35-39] is actually *D. melanica*; M. Pfrender, unpublished data). Because of its large body size (up to 4 mm) and dark pigmentation, this conspicuous species is particularly vulnerable to introduced fish predators. However, in a subset of lakes non-native fish and *Daphnia *coexist [36], providing a unique opportunity to study the evolutionary consequences of introduced fish predators.

This study reports the phenotypic patterns and rates of divergence in naturally occurring populations of *D. melanica *coexisting with introduced fish predators. Several studies have reported rapid adaptive changes in native prey populations following the introduction of novel predators. However, few studies report estimates of the rate of adaptation or divergence in conjunction with observed patterns. Quantitative estimates of evolutionary rates facilitate comparisons across studies, traits, taxa, and time frames [40]. Because we estimate rates of divergence in prey populations exposed to novel predators, we discuss our results in the context of other studies that have examined patterns of divergence, as well as those studies in which evolutionary rates were estimated.

## Results

### Patterns of divergence

**Figure 1:**
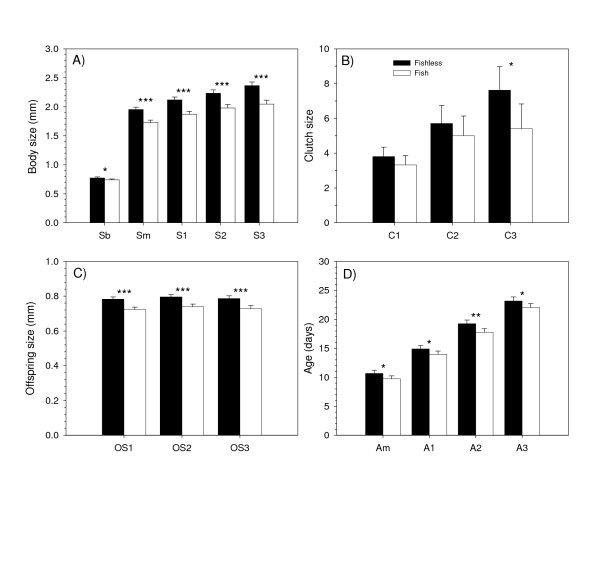
**Bar graphs of morphological and life-history trait means *Daphnia *populations in fishless and fish lakes**. Fishless populations are shown in black and populations co-occuring with fish in white. A) body sizes; B) clutch sizes; C) offspring sizes; D) instar-specific ages. Bars represent the mean with error bars = 2SE. Significance levels for differences in trait means generated by a NANOVA (* = p < 0.05; ** = p,0.01; *** = p < 0.001). See text for further explanation of trait designations and analysis.

Due to some *Daphnia *mortality during the course of the experiment, sample sizes varied among traits (Table 1). Fish and fishless populations differed significantly for all instar-specific body sizes, instar-specific offspring sizes, and age traits measured (Table 1 and Fig. 1A,C,D). *Daphnia *from fishless lakes were significantly larger at birth, matured at a larger body size, and produced larger offspring than *Daphnia *from lakes containing fish. Fecundity did not differ between fish and fishless lakes until release of the third clutch, at which point fishless populations produced significantly more offspring (Table 1 and Fig. 1B). Age at maturity and all subsequent instar-specific ages were significantly longer in fishless populations than fish populations (Table 1 and Fig. 1D). No significant differences were observed between fish and fishless populations for age-specific estimates of growth rate (Table 1).

**Table 1:** Effects of introduced fish on morphological and life-history traits.

	Lake type				
Trait	Fishless (SE)	Fish (SE)	N	F	p-value

S_b_	0.770 (0.010)	0.740 (0.008)	121	5.33	**0.0228**
S_m_	1.952 (0.021)	1.728 (0.021)	120	58.40	**< 0.0001**
S_1_	2.119 (0.025)	1.872 (0.025)	116	47.90	**< 0.0001**
S_2_	2.234 (0.028)	1.981 (0.030)	102	39.12	**< 0.0001**
S_3_	2.363 (0.033)	2.048 (0.032)	82	45.73	**< 0.0001**
C_1_	3.802 (0.261)	3.318 (0.272)	111	1.65	0.2015
C_2_	5.707 (0.522)	4.999 (0.569)	93	0.84	0.3621
C_3_	7.615 (0.680)	5.408 (0.712)	81	5.03	**0.0279**
OS_1_	0.782 (0.007)	0.723 (0.007)	109	34.85	**< 0.0001**
OS_2_	0.795 (0.007)	0.739 (0.008)	92	26.63	**< 0.0001**
OS_3_	0.786 (0.008)	0.728 (0.009)	79	24.15	**< 0.0001**
A_m_	10.676 (0.277)	9.741 (0.256)	108	6.15	**0.0148**
A_1_	14.893 (0.300)	13.963 (0.282)	105	5.10	**0.0261**
A_2_	19.246 (0.320)	17.748 (0.311)	93	11.29	**0.0012**
A_3_	23.152 (0.362)	22.042 (0.334)	77	5.08	**0.0274**
GR_j_	0.090 (0.003)	0.088 (0.003)	107	0.31	0.5801
GR_1_	0.020 (0.002)	0.019 (0.002)	116	0.11	0.7359
GR_2_	0.013 (0.001)	0.014 (0.002)	100	0.12	0.7289
GR_3_	0.010 (0.002)	0.010 (0.002)	78	0.00	0.9916
GR_a_	0.012 (0.001)	0.012 (0.001)	81	0.00	0.9916

Results of one-way mixed model NANOVAs with lake type (fishless and fish) and population nested within lake type as fixed effects. Least-squared means and associated standard errors are given for 19 morphological and life-history traits related to body size, clutch size, instar-specific ages, and instar-specific growth rates (see text for explanation of the trait designations). Number of observations (N), F statistic (F) and p-values for the main effect of lake type (significant values in bold).

### Patterns of character change

**Figure 2:**
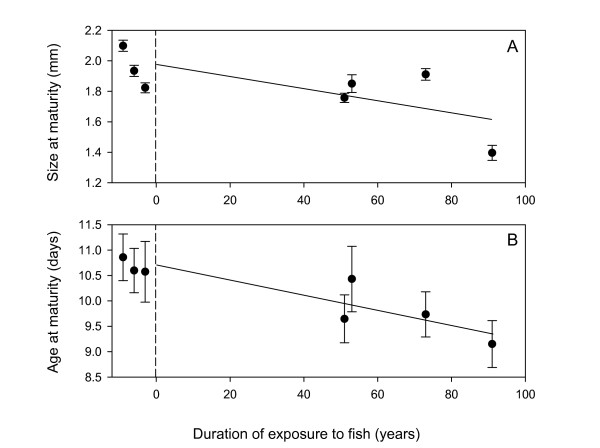
**Trait means versus time exposed to fish predation**. Linear regressions of body size (A) and age at maturity (B) versus the number of years co-occurring with introduced salmonids. Points left of the dashed line are lakes that have never contained introduced salmonids. These points have been offset to the left for visualization. The regression analysis treated these three points as zero years of co-occurrence with fish.

Significant linear regressions of trait means on exposure time to fish predation (years) were obtained for all characters showing divergence based on NANOVA. The rate of decline for size at birth in response to exposure to fish predation was 0.0008 mm/year (Table 2). The rate of decline in body size at maturity was 0.0042 mm/yr (Fig. 2A). Averaged over all instars, the decline in body size was approximately 0.005 mm/year, while offspring size was reduced on average by 0.0012 mm/year. Age at maturity declined by 0.015 days/year (Table 2 and Fig. 2B) and the average instar duration was reduced by 0.018 days/year.

**Table 2:** Summary of the results of linear regressions of trait values versus years with introduced fish.

Trait	Slope (SE)	P-value	*r*^*2*^
S_b_	**-0.0008 (0.0002)**	**0.0030**	**0.104**
S_m_	**-0.0042 (0.0005)**	**< 0.0001**	**0.337**
S_1_	**-0.0048 (0.0006)**	**< 0.0001**	**0.331**
S_2_	**-0.0052 (0.0008)**	**< 0.0001**	**0.299**
S_3_	**-0.0060 (0.0009)**	**< 0.0001**	**0.332**
C_1_	-0.0106 (0.0054)	0.0535	0.034
C_2_	-0.0222 (0.0114)	0.0538	0.040
C_3_	**-0.0378 (0.0149)**	**0.0132**	**0.075**
OS_1_	**-0.0013 (0.0002)**	**< 0.0001**	**0.340**
OS_2_	**-0.0011 (0.0002)**	**< 0.0001**	**0.255**
OS_3_	**-0.0011 (0.0002)**	**< 0.0001**	**0.250**
A_m_	**-0.0151 (0.0052)**	**0.0045**	**0.074**
A_1_	**-0.0153 (0.0056)**	**0.0081**	**0.066**
A_2_	**-0.0222 (0.0060)**	**0.0004**	**0.131**
A_3_	**-0.0163 (0.0069)**	**0.0209**	**0.069**
GR_j_	-0.0000 (0.0001)	0.6024	0.003
GR_1_	-0.0000 (0.0000)	0.5092	0.004
GR_2_	0.0000 (0.0000)	0.9233	0.000
GR_3_	-0.0000 (0.0000)	0.7199	0.002
GR_a_	-0.0000 (0.0000)	0.6626	0.002

The table shows the  slope of the regression of trait value versus duration of exposure to  fish predation (SE), the significance of the slope differing from zero,  and the coefficient of determination. Significant results are shown in bold. See text for trait designations.

### Rates of divergence

Evolutionary change based on absolute time scales yield rates of divergence that are, on average, higher for adult size-related traits (mean = 1830 darwins) than for juvenile size-related traits (1027 darwins) and age-related traits (1047 darwins). These results are in slight contrast to rate estimates using haldanes, where average changes in body size were similar for both juveniles and adults (adults: 0.016 haldanes, juveniles: 0.014 haldanes), and both were higher than rate estimates for timing of reproductive events (0.008 haldanes). Evelyn Lake *Daphnia *show the highest divergence rate for all size-related traits while Lower Goethe has the highest divergence rate for age-related traits (Table 3).

**Table 3:** Divergence rates in response to fish introductions.

			Trait	
Population	Exposure Time (yrs)	Adult Sizes	Juvenile Sizes	Age

Lower Goethe	51	2032 (70)	145 (118)	1490 (198)
		0.019 (0.001)	0.002 (0.002)	0.011 (0.001)
Puppet	53	721 (109)	776 (114)	515 (169)
		0.006 (0.001)	0.010 (0.002)	0.005 (0.002)
Golden	74	330 (82)	576 (104)	993 (128)
		0.003 (0.001)	0.009 (0.002)	0.008 (0.001)
Evelyn	91	4238 (306)	2610 (130)	1188 (204)
		0.034 (0.002)	0.036 (0.003)	0.009 (0.001)

Average estimates of divergence rates in four populations exposed to nonnative salmonid fish based on darwins (upper value) and haldanes (lower value) for three trait types. Means and standard errors (in parentheses) were obtained by averaging over the age-specific estimates for the trait type.

## Discussion

Species introductions can precipitate rapid evolutionary responses in native species [12,41,42]. Several criteria have been proposed to identify cases of rapid adaptive evolution [5]. Among the most important criteria are directional selection with a known cause, an additive genetic basis for traits under selection, and that the response to selection is likely adaptive. We believe our study adequately addresses all of these elements. First, size-selective predation by fish imposes strong directional selection on populations of *Daphnia *[16,22,23,43], and in Sierra Nevada lakes the most conspicuous change in the selective environment is the recent introduction of salmonid fishes. Second, because we utilized a common garden experimental design, observed differences among our populations are genetic in nature. Furthermore, traits under selection in this study are known to show significant heritabilities in other species of *Daphnia *[44,45] and often have substantial levels of additive genetic variation [28]. Finally, the phenotypic response to directional selection is likely to be adaptive because changes we observed are consistent with expectations for populations under size-selective predation. Also useful in identifying rapid evolution is knowledge of the time scale in which the change has taken place and measures of character states before and after the selection event. Because the history of fish introductions is well documented in the Sierra Nevada we know the date of the initial fish stocking in our experimental lakes and the duration of exposure to altered predation regimes. Since this study is cross-sectional and not a temporal series we do not know with certainty what the *Daphnia *phenotypic states in the fish lakes were prior to fish introductions and subsequent selection. However, given that the three fishless lakes we examined did not differ from one another phenotypically, our assumption that phenotypic trait values from populations that have never been exposed to introduced salmonids are representative of trait values in populations prior to fish introductions is justified.

Our results show that introductions of novel fish predators are associated with a specific pattern of phenotypic change in *Daphnia *populations from alpine lakes in the Sierra Nevada. In comparison to historically fishless lakes, *D. melanica *that co-occur with introduced fish are smaller, have smaller offspring, reach maturity earlier and have shorter adult instar durations. Our results are in agreement with other studies showing that the introduction of non-native fish into fishless habitats produces a predictable pattern of rapid divergence from populations not containing fish (*e.g*., [13]).

The reduced body size observed in *D. melanica *populations that co-occur with introduced salmonids is consistent with other empirical observations of zooplankton responses to size-selective vertebrate predation. At the community level, size-selective predation by fish results in a shift in species composition such that smaller bodied species become numerically abundant. This shift in species composition can lead to a decrease in maximum zooplankton body length in as little as 20 years [22]. Furthermore, examination of trout stomach contents show they not only contain a higher proportion of larger-bodied zooplankton species within a lake, but that trout preferentially feed upon genotypes conferring relatively larger body sizes within populations of vulnerable species[23]. The effect of size selective fish predation within a population can manifest over quite short periods of time. For example, mean body size decreased when *Daphnia *were exposed to fish predation for only 50 days [44].

In addition to changes in body size, introductions of fish into naive *Daphnia *populations of the Sierra Nevada also resulted in changes in life-history strategies. Populations exposed to fish predation mature earlier and had fewer, smaller offspring in each clutch than populations from fishless lakes. The changes in clutch size and timing of reproduction are not surprising since these traits frequently co-vary with body size in *Daphnia *[46]. Smaller *Daphnia *tend to carry fewer and/or smaller offspring [47-49].

**Figure 3:**
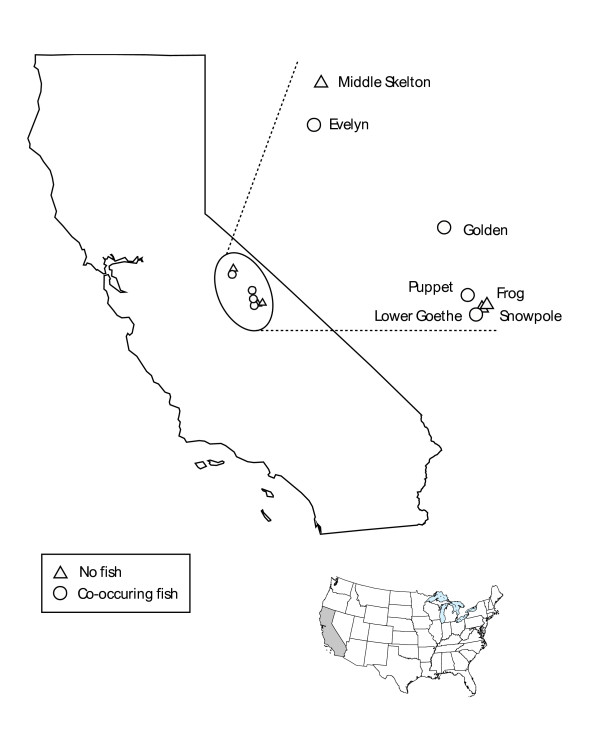
**Map of collection sites in the Sierra Nevada of California, USA**. Sites with no history of salmonid introductions are denoted by (△) and sites with a known history of introductions and resident fish populations by (○).

While we observed a significant correlation between trait divergence and the duration of exposure to fish (Fig. 3) there was considerable variation among lakes in the degree of apparent evolutionary response. The variation in divergence among populations may be due to differences in the initial levels of additive genetic variation or in differences in the intensity of selection. The intensity of selection can greatly affect the rate at which adaptive changes occur. In these alpine lakes differences in the intensity of selection could be the result of varying levels of spatial heterogeneity and fish density among lakes. The relationship between fish density and the intensity of selection has been demonstrated by Cousyn *et al*. [16] who utilized comparisons across a temporal series of *Daphnia *resting eggs collected from lake bottom sediments to show that average body size of *Daphnia *decreased when fish were most dense and increased when fish were least dense.

Our estimates of divergence rates for body size and timing of reproduction are several times to an order of magnitude lower than those obtained in a comparable study of the evolutionary response of Trinidad guppies to introduced fish predators [13]. One explanation for the lower observed rates is that much of the evolution in these populations occurred shortly after fish introductions, resulting in overestimates of the actual time during which evolution actually took place. The possibility of protracted sampling would then result in underestimates of the actual rate of phenotypic change. A second possibility for this observation is that the strength of selection in the Sierra Nevada *Daphnia *populations is not as intense as in the Trinidad guppy system. There are also a number of features of the biology of *Daphnia *that may contribute to a slower response to selection. Since *Daphnia *in these populations hatch from an "egg bank" [50] of diapausing eggs in lake bottom sediments there is a continual input of genotypes from earlier time periods. Genotypes from these earlier time periods may have never been exposed to the novel selective regime or had genotypic values that had not advanced as far in response to selection. The effect of this temporal mixing of genotypes would be to retard the realized rate of evolution [51]. In addition, our estimates of the rate of adaptation is based on the simplifying assumption that one calendar year is equal to one generation. For temporary pond populations of *Daphnia *this is a reasonable assumption since these populations typically engage in a bout of sexual reproduction every year. However, for permanent lake-dwelling populations of *Daphnia *the reproductive cycle can be more complicated. Lake-dwelling populations may engage in sexual reproduction at highly irregular intervals with clones persisting for long periods of time via asexual reproduction. If the Sierra Nevada populations have engaged in sexual reproduction less frequently than on a yearly cycle our estimated rates would be lower than the actual per generation rates. Alternation of asexual and sexual reproduction may also influence the response to selection in two additional ways. First, a cyclic parthenogenetic mode of reproduction can induce oscillations of expressed genetic variation with low levels of expressed variation during periods of prolonged asexual reproduction punctuated by periods of enhanced expressed genetic variation immediately following a bout of sex [52]. During periods of reduced expressed genetic variation populations would respond to phenotypic selection slowly. Second, the response to selection during periods of asexual reproduction can induce substantial gametic phase disequilibria (GPD; non-independence of allele identities at two or more loci). Sexual reproduction and recombination erodes GPD and, assuming an additive genetic basis to the trait, causes a shift in the mean phenotype in the opposite direction from that promoted by selection. This phenomenon is often referred to as genetic slippage and has been observed in a number of populations of *Daphnia *[28,53,54].

## Conclusion

Previous work in the Sierra Nevada has shown that introduced fish have caused many *Daphnia *populations to go locally extinct [36-38]. Our study shows that extant *Daphnia *populations subjected to size-selective fish predation have divergent life-history and morphology compared to populations without fish. The divergence in lakes containing fish and *Daphnia *is in a direction consistent with adaptive evolution and occurred in less than 100 years. This study suggests that native species in aquatic systems are qualitatively similar to terrestrial species in their ability to respond through evolutionary change [43,44]. In light of our results we suggest the rate of evolutionary response and limits to the degree of response will be important determinants of persistence of native populations when faced with selective challenges arising from species introductions, and over the long-term will have a profound influence on community structure.

## Methods

### Specimen collection

*Daphnia melanica *used in this study were collected from seven permanent lakes in the central Sierra Nevada (Fig. 3). These lakes are located in the Humphreys, French Canyon, Upper Mono Creek, and Vogelsang basins at elevations ranging from 3150–3632 meters. Lakes sampled were chosen based on the results of zooplankton sampling [37,38]. Three of these lakes have no history of salmonid introductions (referred to as "Fishless" populations): "Snowpole" (UTM Zone 11: 350430 E, 4123368 N); "Frog" (UTM Zone 11: 351079 E, 4124432 N); and Middle Skelton (UTM Zone 11: 298120 E, 4201298 N). The remaining four lakes have a known history of salmonid introductions including rainbow trout (*Oncorhynchus mykiss*), goldent trout (*O. m., aguabonita*), brown trout (*Salmo trutta*), and brook trout (*Salvelinus fontinalis*) and contain resident populations of *Daphnia *(referred to as "Fish" populations): Lower Goethe (UTM Zone 11: 349094 E, 4121242 N; fish first introduced in 1953); Puppet (UTM Zone 11: 346277 E, 4127817 N); Golden (UTM Zone 11: 343870 E, 4146114 N); and Evelyn (UTM Zone 11: 295393 E, 4186659 N). Based on California Dept. of Fish and Game unpublished stocking records, Lower Goethe Lake has been stocked with golden trout every other year since 1953. Puppet Lake has been stocked with golden trout every other year since 1951. Golden Lake was first stocked with golden trout in 1931 and restocked every other year until 1996. The resident golden trout population is self-sustaining. Evelyn Lake was initially stocked with brown trout in 1913. Brook trout were introduced in 1928, 1946, 1947, 1949, 1951, 1954 and 1958, and rainbow trout were introduced in 1939, 1942, 1944, 1957, 1962, and 1966. No stocking has occurred since 1966, and the resident rainbow trout population is self-sustaining.

*Daphnia *were collected from the study lakes using a 30 cm conical tow net and transported to the laboratory where they were maintained at 4°C for a period of 1–2 weeks. To maximize the amount of genetic variation captured from each population, mature females from the original field collections were then isolated and placed singly in 250 mL beakers containing 200 mL of filtered well-water. Since field collected individuals often carry asexually produced clutches in their brood pouch, this procedure ensured that no isolates were genotypically identical individuals released during the period from collection in the field until isolation in the lab. At 4°C, asexual offspring released prior to isolation in the lab would not have sufficient time to reach maturity. Stock cultures were established from these isolated individuals by clonal reproduction under constant conditions of temperature (15°C) and photoperiod (16L:8D). *Daphnia *were fed a vitamin-supplemented, pure culture of the green alga *Scenedesmus obliquus *every 3–4 days.

### Morphological and life-history trait assay

Morphological and life-history characteristics were assayed using a standard experimental design [28,55]. Briefly, single immature females were used to establish experimental lines from the stock cultures. These lines were then maintained by transferring a single, asexually-produced progeny to a new culture for two generations. Morphological and life-history measures were assayed on third generation individuals. This design minimizes the contribution of maternal and grand-maternal effects to the among-genotype component of variance [56]. In total, twenty randomly chosen clonal lines from each lake were used in the assay, yielding a total of 140 lines (7 lakes × 20 lines).

Individuals in the experimental assay were maintained in 250 mL beakers containing 150 ml of filtered well water supplemented with *S. obliquus *at a concentration of 135,000 cells/mL. The water and algae mixture was replaced every other day to ensure a constant food density. Beakers were randomly assigned to trays and kept in a temperature-controlled chamber at 18°C with a photoperiod of 16L:8D. The position of trays in the chamber was changed every day to minimize micro-environmental differences.

We measured a suite of 20 morphological and life-history traits. Body-size measurements, from the top of the head to the base of the tail spine, were taken using a stereomicroscope and an optic ruler calibrated using a micrometer slide. Size was measured at birth (S_b_), maturity (S_m _– defined as the first instar with deposition of eggs into the brood pouch), and for three successive adult instars following maturity (S_1_, S_2_, S_3_). The number of offspring produced from each of the first three clutches was counted (C_1_, C_2_, C_3_). Clutch-specific estimates of the average body size of offspring were calculated by averaging size measures from four newborns (OS_1_, OS_2_, OS_3_).

During size measurements, the developmental stage of embryos in the brood chamber of mature adults, date, and time were recorded to refine estimates of the timing of maturity (A_m_) and adult instar durations, (A_1_, A_2_, A_3_) [45,58]. The estimated time of reproductive events was then used to calculate four instar-specific estimates of growth rate: growth rate from birth to maturity (GR_j_); from maturity to release of first clutch (GR_1_); the interval between the release of first and second clutch (GR_2_); and the interval between the release of second and third clutch (GR_3_). A measure of mean adult growth rate (GR_a_) was calculated by averaging GR_1_, GR_2_, and GR_3_.

### Data analysis

#### Patterns of divergence

Differences in morphological and life-history traits between *Daphnia *from fish and fishless lakes and among populations were assessed using nested analysis of variance (NANOVA) in which covariance parameters were estimated using restricted maximum likelihood [PROC MIXED; [57]]. Lake type (fish or fishless) and population nested within lake type were treated as fixed effects. We report unadjusted p-values and set our level of significance at p < 0.05. We do not adjust our level of significance based on the number of comparisons (20), but instead use conservatism in our interpretation of results.

#### Patterns of character change

We estimated the nature of character change for each trait with linear regression models using years exposed to fish predation as the independent variable and trait values as the dependent variable [57].

#### Rates of divergence

We calculated trait-specific rates of divergence for each of the four fish populations using haldanes and darwins [40,58]. Haldanes are appropriate for comparisons across diverse traits and taxa because the change in means is scaled by the pooled standard deviation, causing them to be independent of trait dimensionality. Darwins represent change on an absolute scale and are influenced by trait dimensionality [59]. We calculated haldanes to facilitate comparisons across traits within our study, and darwins, which are more commonly used than haldanes, for comparison with other studies. For both measures, the population-mean trait values and variances at time 0 were estimated by pooling all three fishless populations (Table 1). Population-mean trait values and variances for time 1 were calculated for each fish population separately. These values were used to calculate rates of divergence between fishless populations and each of the four fish populations. Our calculations of haldanes assume one sexual generation is equivalent to one year, although a sexual generation in *Daphnia *may span several generations of clonal reproduction.

## Authors' contributions

DLF designed and conducted the experiment, contributed to fieldwork to collect the experimental populations, performed statistical analyses, and drafted the manuscript. LCL performed statistical analyses and contributed to manuscript preparation. RAK provided background information on *Daphnia *populations, assisted with fieldwork in the Sierra Nevada, and helped draft the manuscript. MEP conceived of the study, conducted fieldwork in the Sierra Nevada to collect the experimental populations, and contributed to analysis and manuscript preparation. All authors read and approved the final manuscript.
